# Reconsidering dependence on life-sustaining treatment as a criterion for assisted suicide: the Italian legal unicum in comparative perspective

**DOI:** 10.3389/fpubh.2025.1657070

**Published:** 2025-09-19

**Authors:** Marco Albore, Letizia Sorace, Sossio Del Prete, Raffaele La Russa, Gianpietro Volonnino, Paola Frati, Giorgio Bolino

**Affiliations:** ^1^Department of Anatomical, Histological, Forensic and Orthopedic Sciences, Sapienza University, Rome, Italy; ^2^Department of Life, Health and Environmental Sciences, University of L'Aquila, L'Aquila, Italy

**Keywords:** assisted suicide, end-of-life, life-sustaining treatments, health legislation, comparative analysis

## Abstract

The legalization of Medical Assisted Voluntary Death, including assisted suicide is spreading worldwide, alongside the recognition of the centrality of the patient’s right to self-determination even in case of therapeutic desistance. In Italy Law-no. 219/2017 has granted patients the option of refusing therapy including life-sustaining treatments even without justification. The present paper offers a critical analysis of the legal-normative aspects and ethical-clinical implications of constraining assisted suicide to dependence on life-sustaining treatments. Reviewing some of the key bioethical-legal pronouncements, we discuss the current Italian system on assisted suicide in which dependence on life-sustaining treatment, even after the recent Constitutional sentences, is still one of the mandatory requirements, despite several critical profiles. Through a literature overview on medical life-sustaining treatments notion, the dependence on them is analyzed and assessed in clinical, bioethical and validity terms as a requirement for access to assisted suicide. From this it appears how dependence on life-sustaining treatment constraint shows overly ambiguous definitional boundaries, with the risk of inhomogeneous interpretations especially in the Italian framework. Interestingly, our comparative analysis reveals that Italy is a global legal unicum among the main international systems regulating Medical Assisted Voluntary Death; which, conversely, tend to target the issue on terminally or irreversibly suffering patients, independently of dependence on life-sustaining treatment.

## Introduction

The global discussion around Medical Assisted Voluntary Death (MAVD), including assisted suicide (AS), is intensifying, with growing calls for legalization driven by the patient’s fundamental right to self-determination. The Italian Law No. 219 of 2017 was made to give patients the right to decide for their care, including the interruption of active therapies and life-sustaining treatments (LST)—even planned in advance—in order to be left with only pain management (up to continuous deep sedation if needed) and progressively lead through the end; and it establishes that such a choice does not conflict with constitutionally protected goods or values (1, 2).

In addition to this, the Constitutional Court’s (CC) Order No. 207/2018 and Sentence No. 242/2019 have fundamentally reshaped the conversation around MAVD and AS in Italy. These key pronouncements signal a major shift across legal, bioethical, and clinical domains, pushing for a new approach to the caregiving relationship (3).

However, these jurisprudential remain cautious, considering the persistence of regulatory vacuum in end-life care in Italy (4). On the other hand, they reveal certain critical aspects. A key contentious point is the requirement of dependence on life-sustaining treatment (DLST) for AS access, which is a *unicum* in the World. This condition stands out as the most problematic aspect.

The Assize Court of Massa (judg. 27.7.2020) first challenged this by suggesting a broader interpretation of LST, a sentiment echoed recently by the GIP of Florence (order n.32/2024), who referred the DLST’s constitutionality to the CC. The lack of a clear definition for DLST has led to several interpretive challenges regarding constitutional mandates, resulting in differing judicial stances over the years. In its recent Sentence No. 135/2024, the Constitutional Court (CC) rejected the proposal to eliminate the DLST requirement. However, it simultaneously endorsed a broader definition of LST, now including procedures that, regardless of their technical complexity or invasiveness, are deemed vital for a patient’s survival, and whose interruption would lead to the patient’s death within a short period. This contrasts sharply with the National Bioethics Committee’s (CNB) recent opinion advocating for a stricter DLST definition (5).

The underlying reason for the court’s judgment is essentially driven by the field of application of L.219/2017 identified by judg. 242/2019, which, ‘*in the absence of legislative intervention”* (7.1 Considered in law), represents an ineliminable reference in the assessment of legitimacy to the AS. The court also took into account the recent pronouncements on Hungarian case law of the ECHR, which, leaving a wide decision-making autonomy to the member countries, did not neglect the role that legal safeguards (such as the DLST) can play in contrasting risk of abuse (6).

Nevertheless, despite these important declarations, a climate of ambiguity still persists on the subject of the end of life, both at international and national level.

Recently, in this absence of specific national Italian legislation, the Tuscany Region passed a law (Regional Law No. 16 of March 14, 2025) to regulate the organizational procedures for accessing Medical Assisted Suicide (MAS). This law, however, faithfully incorporates the four stringent requirements for MAS access set forth by the Constitutional Court (in Sentences 242/2019 and 135/2024), including the dependence on life-sustaining treatments (7).

It is crucial to stress that the CC has consistently maintained its role is not to usurp the legislature’s power in balancing the right to self-determination against the right to life. Instead, it merely sets the minimum constitutionally required guarantee given the current legal landscape, leaving ample room for future legislation to devise solutions offering stronger protections for either right.

## The unique italian *vulnus*: comparative literature review

### Clinical declinations of life-sustaining treatments

The term LST boasts international use as interventions mostly aim to support major organ functions and prolonging life without reversing underlying medical conditions (8–11): mechanical ventilation, cardiopulmonary resuscitation, vasopressor drugs, dialysis, artificial hydration-nutrition (12–15). Other measures that can be considered LST: antibiotics, hemotransfusions, NPPV, pacemakers (16–18).

The demarcation line between LST and curative-treatments is not always sharp, but mostly it is fairly noticeable. However, definition of LST can be extended to other therapies, which strictly speaking cannot be termed ‘vital’ as not directly necessary to vital functions but nevertheless essential for survival and enabling a life quality otherwise felt unsustainable: bladder-catheterization, bowel-evacuations, bronchial-aspirations, chemo-radio-therapies; sedation massive analgesia (19–24).

Thus, depending on the individual case’s interpretation, it is a constraint capable of greatly limiting the AS casuistry or, conversely, demolishing its boundaries so much that it is almost all-encompassing.

In the Italian system, LSTs are mentioned in the art.1 par.5 Law 219/2017, where rationale is different: even for diseases in the presence of which that type of treatment is (or should be) in place, in patients destined to live, its interruption, abstention or withdrawal, with explicit lethal effects, are permitted. Indeed, to renounce LSTs ex L.219/2017, the severity and irreversibility indicated by the CC are not required since patient’s judgment of therapeutic disproportionality is broader and non-questionable.

### Law 219/2017 and informed consent

L.219/2017, which regulates informed consent (IC) and advance treatment directives (ATD), represents a milestone in the Italian legal system regarding patient rights. It has introduced a clearer and more articulated regulatory framework than previous legislation, focused on respect for the autonomy and dignity of the patient. A particularly delicate aspect of the law concerns the possibility to interrupt LST in situations of serious and irreversible illness, connected to the patient’s ability to make an informed and voluntary decision. In this context, the assessment of psychological requirements is essential to ensure that the consent given is actually valid.

L.219/2017 establishes that any treatment, including refusal of LST, must be preceded by the patient’s IC. IC implies that the patient is adequately informed about the characteristics and effects of treatments as well risks, alternatives, and consequences of any refusal.

A fundamental requirement for IC, both in the case of refusal and acceptance of treatments, is the patient’s psychic ability to make decisions independently, consciously and voluntarily. In other words, the patient must be able to understand the information provided to him and to evaluate the implications of his choices. Psychic capacity is a broad concept that includes several aspects:

- Understanding: ability to understand informations received about his condition, the treatment options and the consequences of his choices. This implies an adequate cognitive function to perceive, memorize and reprocess information relating to one’s health situation.
- Assessment skills: capability to evaluate the available options in a thoughtful way, understanding the implications of his decisions. For example, when deciding to discontinue LST, the patient must be able to consider alternatives, such as continued treatment and its consequences, and make a choice that reflects his or her values, desires and beliefs.
- Ability to express will: capacity to express decisions freely and not influenced by external factors, pressure or manipulation.

The law does not provide specific criteria for the assessment of psychic capacity, leaving room for interpretation and medico-legal practice. The doctor is responsible for verifying that the patient meets the necessary requirements for an IC. This evaluation includes several phases:

1. Interview, information: the doctor is required to provide the patient with all the necessary information in a clear, understandable and appropriate way to the situation, taking into account any linguistic or cognitive difficulties. In the case of serious illness, such as in situations where a decision is required to stop LST, the doctor will also have to consider the emotional state of the patient, which could affect his or her ability to understand.
2. Mental condition check: If the patient seems unable to understand or evaluate information due to a psychic, cognitive, or emotional disorder (e.g., severe depression, confusion, unconsciousness), the physician should consider the need for psychological or psychiatric support to help him make an informed choice. In some cases, it may be necessary to involve a psychologist or psychiatrist for specialist advice.
3. Autonomy, freedom of choice: patient must be free from external or coercive influences at the time of decision. In some situations, such as in older adults with neurodegenerative diseases or in vulnerable people, it may be necessary to ensure that there are no social pressures. If the decision is suspected to be influenced by external factors, the doctor may decide to have a further psychological evaluation.
4. Possibility of reviewing the decision: The person must have the opportunity to review their decision, especially if the psychological or physical condition changes. If there are doubts about the stability of the decision, it is useful to have a review process that allows you to monitor the evolution of the patient’s thinking

In the case of mental incapacity-incapacitation, the role of the family and legal representatives is crucial. L.219/2017 allows patients to designate a trustee to express his wishes in situations where he is no longer able to do so independently. This trustee, freely chosen, must be a trusted person who reflects his values and preferences, and is called upon to respect the patient’s decisions, even when these concern LST refusal.

If a person is unable to give IC due to a severe mental-cognitive infirmity (e.g., dementia, vegetative-state), the law provides that the decision must be made by a guardian or trusted person, but always taking into account the patient’s previously expressed wishes, where possible. In the absence of ATD or designated trustee, the doctor is required to consult family and legal representatives, trying to respect the patient’s wishes, even in the presence of cognitive limitations.

From a medico-legal perspective, LST interruption is a complex issue that raises several ethical-juridical questions:

1. Relevance of IC: In a medico-legal context, IC represents a fundamental pillar. It is essential that the person has been adequately informed about risks, implications and alternatives.
2. Written documentation and witnesses: Any decision to stop treatment must be properly documented, with all relevant details being collected. This includes recording medical consultations, discussions with family members, and documenting patient consent. In some cases, the presence of witnesses is necessary, as in the case of people unable to express themselves independently.
3. Mental integrity check: In the event of litigation or legal uncertainty, the psychiatric evaluation of the patient becomes crucial. Legal and medical professionals must be able to prove that the person was psychologically fit to make an IC, without any distortion of judgment due altered mental conditions.
4. Physician Responsibilities: Physicians must act in compliance with applicable regulations and ethical guidelines. They must ensure that the interruption of treatments only takes place when all legal and psychological conditions are met, minimizing the risk of legal action for negligence or abuse.

Hence, it is clear from the above that leading subject of L. 219/2017 is care relationship and not MAD, which is only touched on tangentially. In this context, the concept of the DLST underlies the defense of patient-self-determination.

Even in cases where LSTs are (or should be) applied to patients who are expected to live, their discontinuation, non-initiation, or withdrawal, leading to explicit lethal effects, is allowed. Crucially, under Law No. 219 of 2017, the patient’s decision to forgo LSTs does not require the severity and irreversibility criteria specified by the Constitutional Court, because the patient’s determination of therapeutic disproportionality is final.

In the current Italian framework, however, DLST also represents a prerequisite for a positive request for aid-to-die by one’s own hand: now we are on the frontiers of right-to-die, far beyond autonomy in health choices.

### The mandatory requirements of DLST and comparative analysis with international systems

The need to maintain DLST requirement, albeit in its extended definition approved by Sentence No.135/2024, seems to remain crucial for CC for protecting vulnerable and fragile situations, which could otherwise be exposed to risks of abuse or direct–indirect persuasive pressures from third parties or social factors. This position is particularly significant when considering the clear difference from other Countries, such as German, Austrian, and Spanish CCs. The Italian CC diverges by arriving at a different evaluative outcome, given the different national context within which the current Italian landscape operates.

Nevertheless, it is unclear how DLST can be a regulatory criterion proportionate to the purpose of protecting frailty (presided by Art.580 Italian Criminal Code). DTSV appears to be completely irrelevant with respect to the coexistence of the other requirements: it does not imply the irreversibility of a disease and related suffering, nor viceversa. At most, it admits the opposite: a patient with a poor prognosis in DLST, but not necessarily determined to die, may be induced to this decision by exogenous influences. It is therefore conceivable to equate patients with all 4 requirements set out by the CC and those—such as terminally ill, neoplastic, neurodegenerative patients—who at a certain point in their medical history do not “benefit” from the LST, often for incidental reasons. The legitimate intention of these patients to die, even if irreversible and forced to endure intolerable suffering, forces them into longer but avoidable periods of agony. This renders the DLST an unsuitable filter in assessing the legitimacy of the AS, completely disproportionate and superfluous to the purpose of protecting the vulnerabilities assigned to it.

These are probably the reasons why DLST is not included globally in any of the many national regulations on AD, such as Netherlands (25–27), Germany (28, 29), Switzerland (30), Belgium (31, 32), Australia (33, 34), USA (35–37), Canada (38, 39), Spain (40, 41), Colombia (42). Conversely, the presence of an incurable and irreversible illness, and unbearable and incurable suffering, represent requirements that, despite some differences, are almost consistently present, both in Italy and in other countries (25, 28, 29, 43–47).

Unlike in Italy, some countries—such as France (48, 49) many US country [Oregon (36), Washington (50), Vermont (51), Montana (52), California (53), Colorado (54), Hawaii (55), Maine and New Jersey (44), Colombia (73), Australia (33, 34), and the recent English Bill (56, 57)]—require the individual to suffer from a terminal illness with a poor and time-limited prognosis. In some cases, this is estimated within a well-defined timeframe, such as at 8 or 6 months, or extended for a longer period in case of neurodegenerative diseases.

In many other countries, such as Belgium (32), the Netherlands (43), and Spain (40), including Italy’s current AS system, access to AS is permitted even without a terminal illness. Instead, it requires the suffering to be unbearable and incurable, stemming from a severe and irreversible medical condition for which there is not a precise time-limited prognosis at the time of the request.

In Switzerland, aid-in-dying is even permitted in all cases where there are no selfish reasons. And since access is allowed even for non-residents (as in few other places, like Oregon), unlike most states where access is limited to residents, this has led to the phenomenon of “suicide tourism” (30).

One of the main Italian paradoxes lies precisely in the absurdity that many terminal patients (oncological) are denied the right AS because they are not (yet) kept alive by LST, although in an extreme physical decline that leads to an inevitable prelude to death. All this occurs despite these patients, regardless of the DLST, are the main protected subjects in foreign AS systems (58–64) and the vast majority of applicants (46, 65–69).

## Medico-legal and ethical discussions

Italy’s end-of-life framework, marked by fragmented jurisprudence and inadequate national law, unacceptably bars irreversibly ill, suffering patients from AS if they are not on LST. This unique DLST requirement paradoxically excludes many terminally ill individuals (e.g., cancer, neurodegenerative patients) who are covered by assisted dying laws elsewhere, regardless of LST.

The definition of LST is inconsistent in literature, and DLST itself appears neither necessary to prevent abuse nor effective in its protective role; instead, it unreasonably stifles valid requests for aid in unbearable situations. The insistence on the DLST appears disproportionate and potentially superfluous in assessing the legitimacy of AS, especially when other stringent criteria (irreversible illness, intolerable suffering, sound mind) are already met.

The realm of AS is based on altruistic-compassionate aims, respecting the wishes and dignity of patients who are in an extreme and irreversibly near-death conditions (28, 46, 70–72). Prolonging the wait for death entails a greater burden of suffering and prejudice to the person’s values, linked not only to the illness, but also to the contemplation of the inevitable final decline that their loved ones may witness. Likewise, it is a possibility that a patient who is now devoid of concrete possibilities for improvement will lean toward requesting AS, specifically in cases where the natural course is perceived as too slow. Besides, it is possible that anti-conservative ideas may be encouraged in those who incongruously are not entitled to be helped-in-dying. These extreme complications outline a frankly macabre scenario, especially if it depends on a legal constraint lacking additional elements of protection or greater functionality of the system, requiring patients to cruelly sacrifice pushing themselves beyond their physiological limits.

While safeguarding vulnerable individuals is crucial, the legitimate defense of life should not disproportionately override the right to individual liberty, especially through ambiguous legal constraints. Therefore, it is time for Italy to move beyond current interpretations of DLST. We need new, relevant solutions that address modern circumstances and reconsider whether this additional—and possibly superfluous—legal constraint truly serves its purpose when all other conditions for AS are met.

In conclusion, it is appropriate to respond to the stimuli from recent jurisprudence by urging new solutions that are historically fitting, contemporary, and aligned with emerging modern legal directives. The question then becomes whether it is reasonable to maintain the DLST requirement for accessing AS, even when all other criteria are fully met.

In light of the bioethical-doctrinal considerations and all the critical issues highlighted so far, we believe it is appropriate that Italy align with international standards through a definitive political directive that can finally protect the legitimate right to a dignified death, free from the unreasonable legal quibble of DLST constraint, based instead on the truly essential aspects of such dramatic personal situations (irreversible illness, intolerable suffering, voluntary decision).

## Data Availability

The original contributions presented in the study are included in the article/supplementary material, further inquiries can be directed to the corresponding author.
